# Allostatic load as a predictor of all-cause and cause-specific mortality in the general population: Evidence from the Scottish Health Survey

**DOI:** 10.1371/journal.pone.0183297

**Published:** 2017-08-16

**Authors:** Tony Robertson, Gayle Beveridge, Catherine Bromley

**Affiliations:** 1 Centre for Public Health and Population Health Research, Faculty of Health Sciences & Sport, University of Stirling, Stirling, Scotland; 2 Scottish Collaboration for Public Health Research & Policy, University of Edinburgh, Edinburgh, Scotland; 3 UK Statistics Authority, Edinburgh, Scotland; Yokohama City University, JAPAN

## Abstract

Allostatic load is a multiple biomarker measure of physiological ‘wear and tear’ that has shown some promise as marker of overall physiological health, but its power as a risk predictor for mortality and morbidity is less well known. This study has used data from the 2003 Scottish Health Survey (SHeS) (nationally representative sample of Scottish population) linked to mortality records to assess how well allostatic load predicts all-cause and cause-specific mortality. From the sample, data from 4,488 men and women were available with mortality status at 5 and 9.5 (rounded to 10) years after sampling in 2003. Cox proportional hazard models estimated the risk of death (all-cause and the five major causes of death in the population) according to allostatic load score. Multiple imputation was used to address missing values in the dataset. Analyses were also adjusted for potential confounders (sex, age and deprivation). There were 258 and 618 deaths over the 5-year and 10-year follow-up period, respectively. In the fully-adjusted model, higher allostatic load (poorer physiological ‘health’) was not associated with an increased risk of all-cause mortality after 5 years (HR = 1.07, 95% CI 0.94 to 1.22; p = 0.269), but it was after 10 years (HR = 1.08, 95% CI 1.01 to 1.16; p = 0.026). Allostatic load was not associated with specific causes of death over the same follow-up period. In conclusions, greater physiological wear and tear across multiple physiological systems, as measured by allostatic load, is associated with an increased risk of death, but may not be as useful as a predictor for specific causes of death.

## Introduction

Allostatic Load (AL) is “the wear and tear on the body and brain resulting from chronic overactivity or inactivity of physiological systems that are normally involved in adaptation to environmental challenge”.[1] This type of physiological wear and tear occurs across multiple physiological systems in the body. The most widely used construct of AL has been developed by Seeman and colleagues, who have conceptualised it using biomarker measures across an array of systems including the cardiovascular, metabolic and inflammatory systems.[2] Typically an individual’s biomarker levels, based on their distribution within the sample used (e.g. falling within a high-risk percentile or using standardised measures such as z-scores), are summed to produce an overall AL score. The use of concepts such as AL to try and better understand how the environments we live in can affect our physiology and health falls under a holistic approach, in contrast to the more reductionist approach often sought in epidemiology. While the reductionist approach has great value, especially in trying to elucidate causal mechanisms underpinned by theory and biological plausibility, this approach can feel somewhat incongruous given the complex milieu in which we live our day-to-day lives. Assessing these biomarkers together as AL helps us to understand the physiological burden on the body imposed by exposure to damaging environmental stressors and potentially assess risk for disease and ill health.

AL has been shown to predict the risk of some major physical and mental health outcomes including heart disease and mortality.[3–6] However, these studies have been limited to a small number of population cohorts, in some cases with low sample sizes and narrow age ranges focused on middle and older age adults, as well as a focus on all-cause mortality alone. There is a need to develop more sensitive risk calculators in epidemiology and healthcare, not just for cardiovascular disease (QRISK, ASSIGN, Framingham), but for a range of conditions. The intention is that early detection will lead to appropriate interventions, which could be treatments and/or lifestyle changes, which will decrease both morbidity and premature mortality.

This study has used a large, nationally representative population study from ages 16 and up (2003 Scottish Health Survey) to investigate the association between AL and mortality after five and ten years. In addition to all-cause mortality, this study has investigated AL’s ability to predict the risk of death for the five major killers in the population (circulatory system diseases, neoplasms, respiratory system diseases, mental and behavioural disorders, and digestive system diseases).[7] Our hypothesis is that increasing AL (greater physiological burden) would predict a greater risk of death by any cause, as well as the five major causes, most notable after ten years of follow-up. In addition, we hypothesise that AL would be a greater risk predictor when compared to the individual biomarkers that make up the measure.

## Methods

Details of reporting for the STROBE checklist are available in S1 Table.

### Survey data

The analysis used data from the 2003 Scottish Health Survey (SHeS) and administratively linked data from the Scottish Morbidity Records (SMR).[8] The SHeS is a repeat cross-sectional study (annual since 2008, with previous waves in 1995, 1998 and 2003), which provides representative data on the Scottish population’s health, for children and adults residing within private households. The 2003 SHeS used a stratified, random probability sample, designed to provide data at both national and regional level. This data is freely available from UK Data Service.[9]

**Fig 1:**
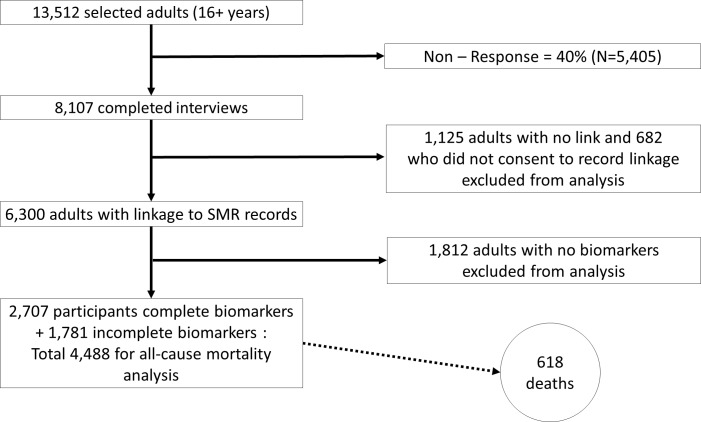
Flowchart of survey response and data linkage.

Participants were asked to consent to have their survey data linked to the SMR data held at the Information Services Division (ISD) of NHS Scotland. SMR data contains details of deaths (including cause) and hospital admissions and is only available upon application to iSD. Fig 1 provides details of the survey response and consent to data linkage. Of 13,512 adults (age 16+ years) selected for the survey, 8,107 completed the first-stage interview, a response rate of 60%. 682 were excluded from the analysis as they did not consent to have their records linked to the SMR and a further 1,125 were excluded as their records were not linked, leaving 6,300 adults with linked records eligible for inclusion in the analysis. Ethical approval for the 2003 survey was obtained from the Multi-Centre Research Ethics Committee for Scotland before fieldwork started.

The SHeS was carried out between June 2003 and December 2004. The actual date of interview was not provided in the dataset due to confidentiality, so 1st June 2003 was used as the start date for all participants in the analysis. The SMR data provided details of deaths up to 31st December 2012, therefore the maximum survival time for the mortality analysis was 3,501 days (9.5 years, but referred to as 10-year risk hereafter). The SMR data includes date of death and primary cause of death as per the death certificate, based on International Classification of Disease (ICD) 10th revision codes.[10] For this analysis, death by any cause was taken at 5 years (31^st^ May 2008) and 10 years (31^st^ December 2012).

### Allostatic load (AL)

AL was constructed from eight biomarker variables, designed to summarise levels of physiological activity across a range of regulatory systems related to disease and mortality risk and used in previous validated AL constructs.[11,12] These included markers across the cardiovascular system (pulse rate, systolic blood pressure (sBP) and diastolic blood pressure (dBP)); the metabolic system (total serum cholesterol (TChol), high density lipoprotein cholesterol (HDL-Chol), waist to hip ratio (WHR) and glycosylated haemoglobin (Hb-A1c); and the inflammatory system (C-Reactive protein (CRP)).

It was assumed that individuals taking medications had already experienced physiological dysregulation [13] so variables were adjusted for the effects of medications to better capture the underlying biomarker values as follows. For anti-hypertensive medications, SBP and DBP were increased by 10mmHg and 5mmHg respectively.[14] For statins, TChol was increased by 1.18 mmol/l.[15] For beta-blockers, HDL-Chol was increased by 10%.[16] For diabetes medication, Hb-A1c was increased by 1%.[17] For diuretics, TChol was reduced by 4%.[16] The results were not substantively altered when using an alternative method (statistical adjustment) for these medications using dummy variables (or alternatively by not adjusting for medications).

AL scores were calculated based on similar methods described elsewhere.[2,11] For each measure of AL, individuals were assigned a value of ‘1’ for each biomarker where their measurement fell in the highest risk quartile for all measurements except HDL-Chol where the lowest quartile corresponds to highest risk. Given this, AL scores could range from 0–8.

### Potential confounders

Age and sex variables were used in the adjusted analyses, as both are independently associated with risk of mortality as well as AL. To adjust for the confounding effect of socioeconomic deprivation, we used the 2012 Scottish Index of Multiple Deprivation (SIMD) in our analyses. The SIMD is the Scottish Government’s official measure of area based deprivation and is based on 31 indicators to provide a comprehensive picture of relative area deprivation.[18] For this analysis, SIMD has been recoded into quintiles, with SIMD1 the most deprived and SIMD5 the least deprived/most affluent.

### Missing data

Of the eight biomarker variables selected to create the AL score, 2,707 individuals had complete data, 1,781 had some data missing and 1,812 had data missing for all eight biomarkers. Analysis of the baseline characteristics of the three groups (Table 1) revealed adults with no biomarker data tended to be younger than the sample average and were more likely to be in the most deprived quintile of the SIMD. Due to the extent of missing data, this group was excluded from the analysis.

**Table 1:** Baseline characteristics of adults (≥ 16 years) from Scottish Health Survey (2003), linked to Scottish morbidity records.

	All Adults (n = 6,300)	Complete Biomarkers (n = 2,707)	Incomplete Biomarkers (n = 1,781)	No Biomarkers (n = 1,812)
**Sex**				
Male	2,752 (45%)	1,215 (45%)	749 (42%)	788 (44%)
Female	3,548 (55%)	1,492 (55%)	1,032 (58%)	1,024 (56%)
**Mean Age (SD)**	51.0 (18.0)	52.6 (17.0)	51.8 (18.2)	47.9 (18.7)
**SIMD**				
Least deprived	1,150 (18%)	560 (22%)	294 (17%)	296 (16%)
2^nd^	1,348 (21%)	630 (23%)	354 (20%)	364 (20%)
3^rd^	1,373 (22%)	626 (23%)	384 (22%)	363 (20%)
4^th^	1,264 (20%)	532 (20%)	373 (21%)	359 (20%)
Most deprived	1,165 (19%)	359 (13%)	377 (21%)	429 (24%)
**Deaths**	845 (13%)	304 (11%)	314 (18%)	227 (13%)

The group (N = 1,781) with incomplete biomarkers were slightly younger, more likely to be male and more deprived than the complete biomarker group. The proportion of deaths was higher (18% vs 11%) than in the complete biomarker group. This group was combined with the complete biomarker group to make a total analysis sample of 4,488.

S2 Table details the number and proportion of values missing for each variable. The distribution of missing values in part reflects the multi-stage survey design with some consenting to measurements taken at the nurse visit (DBP, SBP, pulse) but refusing the blood sample (TChol, HDL-Chol, CRP, Hb-A1c). The overall proportion of missing data was 20%.

S3 Table details the biomarker ranges and quartile cut-points for each biomarker. For most biomarkers, the highest quartile of risk scores were either lower or similar to clinical cut-points, except for CRP (≥4.1 vs clinical cut-point of ≥3.0mg/L) and total cholesterol (≥6.4 vs ≥5.2). For CRP there were 166 individuals with values above 10mg/L and 17 with values over 40mg/L. Such high levels can be representative of an acute bacterial infection. Sensitivity analysis excluding these 17 highest risk individuals did not alter the results below.

### Multiple imputation

The Multiple Imputation (MI) procedure in Stata version 14 (StataCorp LP, College Station, Texas, USA) was used to impute values for the missing data. MI originated with Donald Rubin and is the method of choice to address problems due to missing values in complex surveys.[19] The MI model included all the variables used in the final analysis plus a further nine that could predict missingness (place of birth, religion, ethnicity, marital status, years of full time education, self-rated health, longstanding illness, self-assessed health and economic activity). The inclusion of such ‘auxiliary’ variables can improve a multiple imputation model through reduction of bias and increased precision where data is considered to be missing at random.[20] Twenty MI datasets were produced as the overall percentage of missing data was 20%.[21] Relative efficiency using 20 imputations was 0.975 and stable with lower (15) and higher (50) numbers of imputations.[22]

### Analysis approach

Cox Proportional Hazard Regression (CPHR) analyses, which take account of the time to events (here, death at 5 and 10 years) were undertaken. Baseline (unadjusted) models (model 1) were estimated first, before additional adjustment for age (model 2), sex (model 3), deprivation (model 4) and finally fully adjusted for age, sex and deprivation (model 5). AL score was treated as a continuous measure in the primary analysis, with further analyses tested treating the score as a categorical variable. Interaction terms were included, but none were significant (p>0.05) and all were removed from the models. To assess whether the cumulative AL measure was more strongly associated with mortality compared to the individual AL components, additional survival analyses were conducted using single biomarkers.

For comparison with results based on multiple imputation, CPHR analyses were also conducted on the complete-case dataset. All analyses were conducted in Stata version 14 (StataCorp LP, College Station, Texas, USA).

## Results

Table 2 provides a breakdown of the main causes of the 618 deaths that occurred in the study sample (N = 4,488) over the 10-year follow-up period (2003 to 2012). Deaths from circulatory system diseases were the most frequent, accounting for 34% (210/618) of all deaths. Neoplasms were nearly as frequent, causing 32% (197/618) of all deaths. Deaths from respiratory causes accounted for another 13% (83/618). The remaining 128 deaths were distributed amongst the other causes, with no single cause amounting to more than 4% of the total. Compared with the top causes of death for Scotland (circulatory system diseases, neoplasms, respiratory system diseases, mental and behavioural disorders, and digestive system diseases) for the same period (2003–2012), the deaths in the SHeS sample were comparable with the Scottish population.[7] Only neoplasms in the SHeS sample were slightly over-represented compared to the whole population (32% vs. 28%), while deaths from mental and behavioural disorders and digestive disorders were marginally under-represented (3–4% vs. 6%).

**Table 2:** Top 5 causes of death in Scotland 2003–2012 compared to 2003 SHeS respondents.

	Whole of Scotland		2003 SHeS respondents	
	Number	Proportion of all deaths	Number	Proportion of all deaths
**Total Deaths**	**553,606**	**100%**	**618**	**100%**
Circulatory system diseases	183,285	33%	210	34%
Neoplasms	155,607	28%	197	32%
Respiratory system diseases	71,258	13%	83	13%
Mental and behavioural disorders	30,929	6%	24	4%
Digestive system diseases	30,778	6%	20	3%
Other causes	81,749	14%	84	14%

Source: National Records of Scotland Vital Events Reference Tables, Table 6.1 (www.nrsscotland.gov.uk)

Table 3 shows the proportion of deaths by age, sex and deprivation for adults aged 16 years and over at baseline in the 5 and 10 year follow-up period from 1^st^ June 2003. Equal proportions of men and women died after both 5 years (2.8–2.9%) and 10 years (6.9%). Deaths increased with age, with 0.1% of 16–24 year olds and 2.9% of 75+ year olds dying after 5 years. By 10 years, deaths in the youngest group had increased in number, but still only represented around 0.1% of the sample, while for the 75+ group the death rate was now at 6.6%. As expected deaths were also higher in more deprived groups, accounting for 1.5% (5 years) and 3.5% (10 years) of the sample.

**Table 3:** Proportion of deaths for all causes during 9.5 year follow-up in adult participants of 2003 Scottish Health Survey aged ≥ 16 years at baseline by age, sex and Scottish Index of Multiple Deprivation (SIMD).

	No. of deaths by 5 years (n = 258)	No. of deaths by 9.5 years (n = 618)
**Sex**		
Men	127 (2.8%)^a^ (49.2%)^b^	310 (6.9%) (50.2%)
Women	131 (2.9%) (50.8%)	308 (6.9%) (49.8%)
**Age at Baseline (years)**		
16–24	2 (0.1%) (0.8%)	5 (0.1%) (0.8%)
25–34	1 (0.1%) (0.4%)	2 (0.1%) (0.3%)
35–44	4 (0.1%) (1.6%)	15 (0.3%) (2.4%)
45–54	13 (0.3%) (5.0%)	28 (0.6%) (4.5%)
55–64	33 (0.7%) (12.8%)	85 (1.9%) (13.8%)
65–74	73 (1.6%) (28.3%)	186 (4.1%) (30.1%)
75+	132 (2.9%) (51.2%)	297 (6.6%) (48.1%)
**SIMD (quintiles)**		
Least deprived	29 (0.6%) (11.2%)	79 (1.8%) (12.8%)
2^nd^	50 (1.1%) (19.4%)	118 (2.6%) (19.1%)
3^rd^	55 (1.2%) (21.3%)	125 (2.8%) (20.2%)
4^th^	57 (1.3%) (22.1%)	141 (3.1%) (22.8%)
Most deprived	67 (1.5%) (26.0%)	155 (3.4%) (25.1%)
**Total**	**258 (5.7%) (100%)**	**618 (13.8%) (100%)**

Percentages are based on

^a^Biomarker eligible sample (n = 4,488 –all adults minus those with no biomarkers)

^b^Death-only sample (n = 258 or n = 618).

In the CPHR analysis, increasing age was associated with an increased risk of death at 5 years (HR = 1.09, 95% CI = 1.08, 1.11, p<0.001) and 10 years (HR = 1.10, 95% CI = 1.09, 1.11, p<0.001). Lower deprivation was associated with a reduced risk of dying, again in both the 5 year (HR = 0.85, 95% CI = 0.77, 0.93, p = 0.001) and 10 year (HR = 0.86, 95% CI = 0.81, 0.91, p<0.001) models. Women had a lower risk of death compared to men after 10 years (HR = 0.84, 95% CI = 0.68, 0.95, p = 0.008), but not 5 years (HR = 0.84, 95% CI = 0.66, 1.08, p = 0.176).

Unadjusted CPHR analysis with multiple imputation (Table 4) showed that there was an association between AL and all-cause mortality, with a one unit increase in AL (i.e. one biomarker being scored in the higher risk quartile) associated with a 46% increased risk of dying from all causes within the 10 year time period (HR = 1.46, 95% CI = 1.38, 1.55, p<0.001). The same association was also seen within 5 years (HR = 1.45, 95% CI = 1.31, 1.60, p<0.001). Focusing on the 5 year model, adjustment for sex, age and deprivation (model 5) attenuated the association, with AL no longer associated with all-cause mortality (HR = 1.07, 95% CI = 0.94, 1.22, p = 0.269). Adjusting for sex and deprivation alone (models 2 and 4) accounted for little of this attenuation, with age the predominant factor as seen in model 3 (HR = 1.10, 95% CI = 0.97, 1.24, p = 0.127). However, in the 10 year analysis, while age once again attenuated the relationship (8% increased risk of death per AL unit), AL remained a significant predictor of all-cause mortality (fully adjusted model 5: HR = 1.08, 95% CI = 1.01, 1.16, p = 0.026). The complete-case analysis for both the 5 and 10-year risk were substantively the same as the imputation models (S4 Table).

**Table 4:** Hazard ratios (HR) for 5 and 10-year risk of death (all-cause) by allostatic load (multiple imputation analysis).

	5 year risk			10 year risk		
	HR	95% CI	p-value	HR	95% CI	p-value
**Model 1**	1.45	1.31, 1.60	<0.001	1.46	1.38, 1.55	<0.001
**Model 2**	1.45	1.31, 1.60	<0.001	1.46	1.38, 1.55	<0.001
**Model 3**	1.10	0.97, 1.24	0.127	1.11	1.04, 1.19	0.003
**Model 4**	1.43	1.29, 1.58	<0.001	1.44	1.36, 1.53	<0.001
**Model 5**	1.07	0.94, 1.22	0.269	1.08	1.01, 1.16	0.025

Where: Model 1: Unadjusted (allostatic load). Model 2: Model 1 + sex. Model 3: Model 1 + age. Model 4: Model 1 + deprivation. Model 5: Model 1 + sex, age and deprivation.

Analyses examining the associations between the biomarkers that make up this AL score and all-cause mortality showed that after controlling for age more negative levels of five of these biomarkers (pulse pressure, HDL cholesterol, WHR, HbA1c and CRP) were associated with an increased risk of death within 10 years (S5A–S5H Table). The magnitude of effect was quite variable though, for example, an increase in HbA1c by one unit (%) was associated with an 18% increased risk of death after 10 years compared to CRP (1mg/L) with only a 2% increased risk. However, diastolic blood pressure and total cholesterol showed the opposite direction of effect, with systolic blood pressure not associated with mortality.

Finally, CPHR analysis against cause-specific mortality (circulatory, neoplasm, respiratory, mental and behavioural, digestive or ‘other’) revealed that allostatic load was not associated with risk of death for any specific cause at 10 years (Table 5).

**Table 5:** Hazard ratio for 10-year risk of death by specific causes modelled against allostatic load unadjusted for potential confounders (multiple imputation).

	10 year risk		
Cause of death	HR	95% CI	p-value
Circulatory	0.99	0.89, 1.09	0.783
Neoplasm	1.04	0.92, 1.18	0.534
Respiratory	0.94	0.76, 1.16	0.548
Mental & Behavioural	0.91	0.68, 1.23	0.537
Digestive	1.00	0.73, 1.35	0.984
Other	0.98	0.84, 1.13	0.741

## Discussion

### Statement of principal findings

This study has found evidence amongst approximately 4,500 adults from the general population in Scotland that increasing allostatic load (AL), a marker of cumulative physiological burden, was associated with an increased risk of all-cause mortality, with a 1 unit increase associated with approximately a 45% increased risk of death by any cause within 5 and 10 years. Adjustment for sex and deprivation had very little effect on this association. However, adjustment for age fully attenuated the relationship in the 5-year model, but remained statistically significant over 10 years with a one-unit increase in AL associated with an 8% increased risk of death. The AL measure was a stronger predictor of mortality than the majority of individual biomarkers used to construct the AL measure (systolic blood pressure, diastolic blood pressure, pulse pressure, total cholesterol and CRP), but is matched by (or exceed by) HDL cholesterol, HbA1c and waist:hip ratio in predicting risk in the fully adjusted 10-year models. Comparisons in terms of effect size are difficult, as the scores are not standardised to the same scale. When investigating the relationship between AL and the top five causes of death in the sample (circulatory, neoplasm, respiratory, mental and behavioural, digestive or ‘other’), AL was not associated with any of the causes investigated. This weakens the case for AL being a useful predictive tool if it cannot predict at the more focused scale where more tailored prevention measures can be trialled.

### Relation to other studies

Six notable studies have investigated the association between AL and mortality, although these have only used data from three cohorts/samples. Borrell et al (2010), Levine & Crimmins (2014) and Howard and Sparks (2016) used data from the third National Health and Nutrition Examination Survey (NHANES III) (1988–1994), a large, nationally representative study of around 40,000 U.S. children and adults (2months old and above). [4,23,24] Borrell et al. examined 12 year mortality in a sample of over 13,000 adults (25 years and above), where almost 2,500 respondents had died. Using a clinical cut-off AL score (that did not contain any neuroendocrine markers), they found that, compared to AL scores of one, those with AL scores of two and three and above had 155% and 429% increased risks of all-cause mortality (unadjusted), respectively. Adjustment for age, sex and ethnicity reduced these risks to 35% and 99%, respectively, with both remaining significant. Further adjustment for socioeconomic factors (education and income) had little further attenuation effect. Levine & Crimmins examined 10-year all-cause mortality with clinical cut-off and z-score AL constructs, but split into quintiles for analysis. They identified that the top AL quintile (clinical cut-offs) had a hazard ratio of 2.75 compared to the lowest quintile (HR = 1) after adjusting for age and sex. They also found that higher AL was associated with an increased risk of death from CVD and cancer, although slightly weaker than with all-cause mortality. The results were matched, although with greater effect sizes (typically double the clinical cut-off), when using the z-score AL score. Investigating mortality over 17 years in NHANES III, Howard and Sparks showed that a one unit increase in AL (using the same construct as Borrell et al) only represented a 7% increased risk of death (adjusted for age, sex, ethnicity, socioeconomic status and health behaviours).

The second study utilised has been the MacArthur studies of successful ageing, a longitudinal study of relatively high functioning men and women aged 70 to 79 years living in the United States (North Carolina, Massachusetts and Connecticut) sampled in 1988/89. Seeman et al (2001) investigated the association between AL and all-cause mortality over 7 years using a sub-sample of 720 people (153 who had died within the follow-up period).[25] Using a quartile measure of AL, containing neuroendocrine markers, they found using logistic regression that a one unit increase in AL was associated with a 23% increased risk of death having adjusted for age, sex, ethnicity, education, income and morbidity at baseline. Karlamangla et al (2006) focused on a smaller sample of 171 respondents (with 19 deaths) who had two measures of AL (in 1988 and 1991) to investigate the effect of changes in AL on mortality risk.[26] Using a z-score derived AL measure, they found using logistic regression that a 1 unit change in AL over 2.5 years was associated with an odds ratio of 3.33.

Hwang et al (2014) used the Taiwanese Social Environment and Biomarkers of Aging Study (SEBAS), a nationally representative longitudinal survey of adults 54 years and older.[5] In a sample of 1,023 (with 177 deaths), a one unit increase in decile-derived AL (with neuroendocrine markers) was associated with a 25% increased risk of all-cause mortality. Adjusting for age and sex reduced the risk to 20%, although it remained statistically significant. As well as all-cause mortality, Hwang et al also investigated specific causes. They found that after adjusting for age and sex, increasing allostatic load was significantly associated with increased risk of death by neoplasm (HR = 1.18), cardiometabolic (HR = 1.20), infectious (HR = 1.21), respiratory (HR = 1.34) and ‘other’ (HR = 1.22) causes.

### Meaning of the study

To the best of our knowledge, this is the first study to investigate the links between AL and mortality in a UK or European population. In common with a small number of studies in US and Taiwanese populations, this paper has identified increased risk of all-cause mortality associated with increasing AL. However, compared to these studies from the USA and Taiwan our adjusted hazard ratio after 10 years (HR = 1.08) is lower than that recorded elsewhere, except for Howard et al’s analysis on the NHANES III sample.(Howard & Sparks 2016) Differences in the biomarkers used to construct AL, for example, the inclusion/exclusion of neuroendocrine markers may explain some of the variation across studies, although there is no clear pattern with or without neuroendocrine markers being included. AL has also been operationalised differently across the studies, with clinical cut-offs (Howard & Sparks 2016; Borrell et al. 2010; Levine & Crimmins 2014), deciles (Hwang et al. 2014), quartiles (Seeman et al. 2001) and z-scores (Karlamangla et al. 2006) used. Different follow-up times have also been used, ranging from 7 to 17 years, as well as a range of ages but largely focused on those in middle and older ages. In addition, various adjustments for confounders/covariates and analysis methods have been utilised across the literature. Given this variety and the small number of studies to date, it is difficult to be sure of the primary reasons for discrepancies between studies, but does highlight the issues across the AL literature. It is clear though that AL remains an independent predictor of all-cause mortality risk that may help provide additional risk prediction and understanding of the links between cumulative physiological burden and health.

As seen previously there are clear links between increasing age and increasing AL, also reflected in our findings.[11,27] Given that AL is a marker of cumulative physiological burden, it is expected that AL would increase with age (building up over time and increased exposure to detrimental experiences and stressors). Given that the SHeS contains people aged 16–95 years old, the effect of age will be more significant than in a cohort of 70 year olds, such as the MacArthur studies.[4] Nevertheless, being able to identify a greater risk for death given higher AL in a population study including all adult ages is important in validating the AL concept and measure so that it is not just relevant to health later in life. Despite this, it is clear from the analyses that AL is likely a blunt instrument in predicting risk, as AL was not significantly associated with death by specific causes. This is in stark contrast to the results from SEBAS, although this analysis did use deciles to estimate AL. These more extreme cut-offs would be more likely to show a stronger association with mortality risk overall, which may partly explain the discrepancy.

### Strengths and weaknesses of the study

One of the strengths of this study was the use of the SHeS dataset, a large nationally representative population survey of Scottish adults (16+). With linkage to mortality records, this provided a prospective follow-up study for analysis across all age groups, both sexes and across socioeconomic groups for approximately 10 years. This study also builds on the limited evidence for the links between AL and mortality and risk prediction. Another strength was the use of multiple imputation for missing data. The use of MI increased the analysis sample size from 2,707 to 4,488, with all the missing variables being biomarkers for use in the AL measure. Compared to the complete-case analysis (S4 Table), the MI analyses have increased statistical power and precision, although the substantive findings were similar in both complete-case and MI analyses. Despite these strengths, we must note some potential limitations.

One potential limitation of this study was the choice of biomarkers used to construct the AL score. Allostatic load theory emphasises the importance of measuring dysregulation across physiological systems. Ideally this would include biomarkers from the cardiovascular, metabolic, immune and neuroendocrine systems.[28] We were limited by the data available and so our AL score did not contain any primary mediators i.e. neuroendocrine biomarkers, such as cortisol. The stress response is believed to play a key role in allostasis and subsequent allostatic load with a series of physiological changes taking place, including in these primary mediators, before initial stress responses occur (‘primary effects’ such as rapid increases in blood pressure and sugars/fats that supply the body with extra energy). These initial responses are followed by secondary outcomes (measured in our allostatic load model) and tertiary (disease) outcomes.[29] However, stress markers are quite difficult to measure, e.g. cortisol shows strong diurnal pattern and significant variation across and within individuals on different days and repeated measurements over 1–2 days are recommended.[30] This makes it difficult to measure in large surveys. Inclusion of neuroendocrine markers could improve the power of the AL score to predict mortality,[2] but their exclusion does not invalidate the AL construct as the cascading effects and outcomes of the neuroendocrine markers are still being included in the measure. However, without directly testing the AL score with and without these measures, it is difficult to estimate the true effect not including such markers would have. Finally, we only focused on one version of operationalising allostatic load, namely the quartile risk method. Reviews of previous studies have identified that there is little variation in predictability of health outcomes based on the method of operationalising allostatic load, although we cannot be certain this is not the case here.[31] Future work could consider the impact of allostatic load operationalisation and the effect on mortality and morbidity risk prediction.

Another limitation is that the biomarker information was only a ‘snapshot’ at a particular time. Greater sensitivity in the prediction of mortality may be achieved by studying the change in biomarker levels (and AL) over time. Finally, there remains the issue of how best to operationalise AL, with the quartile construct used here. The quartile AL score is the most commonly used (given its relative ease to calculate and interpret), but it does suffer from potential issues around oversimplification (by reducing risk to an arbitrary binary score for each biomarker) and being population-specific (based on quartile of risk within the population sample being analysed). This restricts the potential for comparing and pooling samples (along with the issue of various biomarkers being included in different studies). Using a method such as clinical cut-offs does provide a more consistent and clinically-relevant measure, but even then clinical cut-offs will differ between populations and this type of measure risks diverting from the original theory that AL represents a sub-clinical dysregulation state.[31,32]

### Policy and practice relevance

A key aspect of improving public health in the UK and elsewhere is through prevention strategies and early detection of morbidities in the general population. One method commonly employed are health screenings, typically focusing on cancer and newborn health. In recent years, the National Health Service in Scotland, as well as England and Wales, have introduced health check-ups for those turning 40 years of age. These check-ups were intended to help provide early risk predictions for major chronic conditions such as heart disease, diabetes and stroke and then provide further support and advice to help manage and reduce these risks.[33,34] These programmes consume large amounts of public resources despite evidence that they are not effective in addressing their primary purpose (the Scottish health check was scrapped within three years of its implementation).[34,35] In this study, and a handful of others, AL has been shown as an effective risk marker for all-cause mortality and in some cases specific causes. However, it would likely be a similarly expensive tool to implement at a population level and would be a rather blunt tool for assessing risk. Combined with the variety of biomarkers and methods for operationalising AL, it would be difficult to introduce consistently across a population. Indeed, allostatic load has been shown to be less effective at predicting mortality risk when compared to biological age and Framingham, throwing further doubt on its practical use as a clinical risk predictor.[4] This paper only focuses on mortality, rather than morbidity. However, early evidence from the AL literature has shown AL’s effectiveness as a predictor for outcomes such as heart disease, cognitive and physical function and psychiatric disorders.[2,3,36] Therefore, it may have value in helping predict a range of morbidities and multimorbidity that other measures such as Framingham are not designed for. What AL does clearly offer is a tool for moving towards a more holistic view of physiological dysregulation, where an individual’s health is viewed across the whole body rather than focusing on one marker, system or condition.

### Conclusions

AL has been shown to be an effective marker of cumulative physiological dysregulation and burden across the body that can also be used as a risk predictor for mortality. However, there remain questions about how AL could be best integrated into practice to help predict risk at an individual level, as well as across populations and with different health outcomes.

## Supporting information

S1 Table STROBE statement—Checklist of items that should be included in reports of observational studies. (DOC)

S2 Table Number and proportion of missing values in analysis sample. (DOCX)

S3 Table Quartile cut-points for each biomarker in the allostatic load score. (DOCX)

S4 Table Hazard ratios (HR) for 5 and 10-year risk of death (all-cause) by allostatic load (complete-case analysis, n = 2,707). (DOCX)

S5 Table A. Hazard ratio for 5- and 10-year risk of death modelled against systolic blood pressure (multiple imputation).
B. Hazard ratio for 5- and 10-year risk of death modelled against diastolic blood pressure (multiple imputation).
C. Hazard ratio for 5- and 10-year risk of death modelled against pulse pressure (multiple imputation).
D. Hazard ratio for 5- and 10-year risk of death modelled against total cholesterol (multiple imputation).
E. Hazard ratio for 5- and 10-year risk of death modelled against HDL cholesterol (multiple imputation).
F. Hazard ratio for 5- and 10-year risk of death modelled against glycated haemoglobin (HbA1c) (multiple imputation).
G. Hazard ratio for 5- and 10-year risk of death modelled against waist:hip ratio (WHR) (multiple imputation).
H. Hazard ratio for 5- and 10-year risk of death modelled against C-Reactive Protein (CRP) (multiple imputation).
(DOCX)
