# Supplementary material for: Allostatic load as a predictor of all-cause and cause-specific mortality in the general population: Evidence from the Scottish Health Survey
Source: PLoS One. 2017 Aug 16;12(8):e0183297. doi: 10.1371/journal.pone.0183297 (PMC5559080; doi:10.1371/journal.pone.0183297)
Supplement: S2 Table — (DOCX) [file pone.0183297.s002.docx]

**S2 Table. Number and Proportion of Missing Values in Analysis Sample.**

| **Biomarker** | **Missing values** | **Proportion of total analysis sample (N=4,488)** |
| --- | --- | --- |
| **Cardiovascular** |  |  |
| Diastolic Blood Pressure | 759 | 17% |
| Systolic Blood Pressure | 759 | 17% |
| Pulse Rate | 759 | 17% |
| **Metabolic** |  |  |
| Total Cholesterol | 1,049 | 23% |
| HDL Cholesterol | 1,049 | 23% |
| HbA1c | 1,073 | 24% |
| Waist:Hip Ratio | 154 | 3% |
| **Inflammatory** |  |  |
| C-Reactive Protein | 1,066 | 24% |
| **Allostatic Load** | 1,781 | 40% |
