# Supplementary material for: Allostatic load as a predictor of all-cause and cause-specific mortality in the general population: Evidence from the Scottish Health Survey
Source: PLoS One. 2017 Aug 16;12(8):e0183297. doi: 10.1371/journal.pone.0183297 (PMC5559080; doi:10.1371/journal.pone.0183297)
Supplement: S3 Table — (DOCX) [file pone.0183297.s003.docx]

**S3 Table. Quartile cut-points for each biomarker in the Allostaic Load score.**

| **Biomarker** | **Range** | **High-risk quartile cut-point** |
| --- | --- | --- |
| **Cardiovascular** |  |  |
| Diastolic Blood Pressure (mmHg) | 39.5 – 132.0 (11.4) | ≥82.0 |
| Systolic Blood Pressure (mmHg) | 66.0 – 216.50 (19.5) | ≥142.0 |
| Pulse Rate (beats per minute) | 21.0 – 148.5 (15.6) | ≥65.5 |
| **Metabolic** |  |  |
| Total Cholesterol (mmol/L) | 2.5 – 11.4 (1.2) | ≥6.4 |
| HDL Cholesterol (mmol/L) | 0.5 – 3.8 (0.4) | ≤1.2 |
| HbA1c (%) | 2.8 – 12.1 (0.7) | ≥5.6 |
| Waist:Hip Ratio (Men) | 0.7 – 1.2 (0.1) | ≥1.0 |
| Waist:Hip Ratio (Women) | 0.45 – 1.09 (0.1) | ≥0.9 |
| **Inflammatory** |  |  |
| C-Reactive Protein (mg/L) | 0.2 – 94.8 (7.1) | ≥4.1 |
