# Supplementary material for: Allostatic load as a predictor of all-cause and cause-specific mortality in the general population: Evidence from the Scottish Health Survey
Source: PLoS One. 2017 Aug 16;12(8):e0183297. doi: 10.1371/journal.pone.0183297 (PMC5559080; doi:10.1371/journal.pone.0183297)
Supplement: S4 Table — (DOCX) [file pone.0183297.s004.docx]

**S4 Table. Hazard ratios (HR) for 5 and 10-year risk of death (all-cause) by allostatic load (complete-case analysis, n=2,707).**

|  | **5 year risk** | | |  | **10 year risk** | | |
| --- | --- | --- | --- | --- | --- | --- | --- |
|  | **HR** | **95% CI** | **p-value** |  | **HR** | **95% CI** | **p-value** |
| **Model 1** | 1.45 | 1.31, 1.59 | <0.001 |  | 1.48 | 1.38, 1.57 | <0.001 |
| **Model 2** | 1.44 | 1.31, 1.59 | <0.001 |  | 1.48 | 1.39, 1.57 | <0.001 |
| **Model 3** | 1.10 | 0.99, 1.24 | 0.083 |  | 1.13 | 1.06, 1.21 | 0.002 |
| **Model 4** | 1.43 | 1.30, 1.57 | <0.001 |  | 1.46 | 1.37, 1.55 | <0.001 |
| **Model 5** | 1.09 | 0.97, 1.22 | 0.160 |  | 1.10 | 1.02, 1.19 | 0.011 |

Where:

Model 1: Unadjusted (allostatic load)

Model 2: Model 1 + sex

Model 3: Model 1 + age

Model 4: Model 1 + deprivation

Model 5: Model 1 + sex, age and deprivation
