# Supplementary material for: Allostatic load as a predictor of all-cause and cause-specific mortality in the general population: Evidence from the Scottish Health Survey
Source: PLoS One. 2017 Aug 16;12(8):e0183297. doi: 10.1371/journal.pone.0183297 (PMC5559080; doi:10.1371/journal.pone.0183297)
Supplement: S5 Table — A. Hazard ratio for 5- and 10-year risk of death modelled against systolic blood pressure (multiple imputation). B. Hazard ratio for 5- and 10-year risk of death modelled against diastolic blood pressure (multiple imputation). C. Hazard ratio for 5- and 10-year risk of death modelled against pulse pressure (multiple imputation). D. Hazard ratio for 5- and 10-year risk of death modelled against total cholesterol (multiple imputation). E. Hazard ratio for 5- and 10-year risk of death modelled against HDL cholesterol (multiple imputation). F. Hazard ratio for 5- and 10-year risk of death modelled against glycated haemoglobin (HbA1c) (multiple imputation). G. Hazard ratio for 5- and 10-year risk of death modelled against waist:hip ratio (WHR) (multiple imputation). H. Hazard ratio for 5- and 10-year risk of death modelled against C-Reactive Protein (CRP) (multiple imputation). (DOCX) [file pone.0183297.s005.docx]

**S5 /A-S5H Tables**

Where:

Model 1: AL

Model 2: AL + sex

Model 3: AL + age

Model 4: AL + SIMD

Model 5: AL + sex + age + SIMD

**S5A Table. Hazard ratio for 5- and 10-year risk of death modelled against systolic blood pressure (multiple imputation).**

|  | **5 year risk** | | |  | **10 year risk** | | |
| --- | --- | --- | --- | --- | --- | --- | --- |
|  | **HR** | **95% CI** | **p-value** |  | **HR** | **95% CI** | **p-value** |
| **Model 1** | 1.02 | 1.02, 1.03 | <0.001 |  | 1.03 | 1.02, 1.03 | <0.001 |
| **Model 2** | 1.02 | 1.01, 1.03 | <0.001 |  | 1.03 | 1.02, 1.03 | <0.001 |
| **Model 3** | 1.00 | 0.99, 1.00 | 0.279 |  | 1.00 | 0.99, 1.00 | 0.680 |
| **Model 4** | 1.02 | 1.02 1.03 | <0.001 |  | 1.03 | 1.02 1.03 | <0.001 |
| **Model 5** | 1.00 | 0.99, 1.00 | 0.205 |  | 1.00 | 0.99, 1.00 | 0.913 |

**S5B Table. Hazard ratio for 5- and 10-year risk of death modelled against diastolic blood pressure (multiple imputation).**

|  | **5 year risk** | | |  | **10 year risk** | | |
| --- | --- | --- | --- | --- | --- | --- | --- |
|  | **HR** | **95% CI** | **p-value** |  | **HR** | **95% CI** | **p-value** |
| **Model 1** | 0.98 | 0.96, 0.99 | <0.001 |  | 0.98 | 0.98, 0.99 | <0.001 |
| **Model 2** | 0.98 | 0.96, 0.99 | <0.001 |  | 0.98 | 0.98, 0.99 | <0.001 |
| **Model 3** | 0.98 | 0.97, 0.99 | 0.003 |  | 0.99 | 0.98, 1.00 | 0.003 |
| **Model 4** | 0.98 | 0.96, 0.99 | <0.001 |  | 0.98 | 0.98, 0.99 | <0.001 |
| **Model 5** | 0.98 | 0.97, 0.99 | 0.003 |  | 0.99 | 0.98, 1.00 | 0.002 |

**S5C Table. Hazard ratio for 5- and 10-year risk of death modelled against pulse pressure (multiple imputation).**

|  | **5 year risk** | | |  | **10 year risk** | | |
| --- | --- | --- | --- | --- | --- | --- | --- |
|  | **HR** | **95% CI** | **p-value** |  | **HR** | **95% CI** | **p-value** |
| **Model 1** | 1.04 | 1.03, 1.04 | <0.001 |  | 1.04 | 1.04, 1.05 | <0.001 |
| **Model 2** | 1.04 | 1.03, 1.04 | <0.001 |  | 1.04 | 1.04, 1.05 | <0.001 |
| **Model 3** | 1.00 | 1.00, 1.01 | 0.465 |  | 1.01 | 1.00, 1.01 | 0.009 |
| **Model 4** | 1.04 | 1.03, 1.04 | <0.001 |  | 1.04 | 1.04, 1.04 | <0.001 |
| **Model 5** | 1.00 | 0.99, 1.01 | 0.600 |  | 1.01 | 1.00, 1.01 | 0.016 |

**S5D Table. Hazard ratio for 5- and 10-year risk of death modelled against total cholesterol (multiple imputation).**

|  | **5 year risk** | | |  | **10 year risk** | | |
| --- | --- | --- | --- | --- | --- | --- | --- |
|  | **HR** | **95% CI** | **p-value** |  | **HR** | **95% CI** | **p-value** |
| **Model 1** | 0.84 | 0.74, 0.97 | 0.015 |  | 0.95 | 0.88, 1.02 | 0.147 |
| **Model 2** | 0.85 | 0.74, 0.98 | 0.021 |  | 0.96 | 0.89, 1.03 | 0.246 |
| **Model 3** | 0.76 | 0.67, 0.87 | <0.001 |  | 0.83 | 0.78, 0.90 | <0.001 |
| **Model 4** | 0.85 | 0.74, 0.97 | 0.019 |  | 0.95 | 0.88, 1.03 | 0.203 |
| **Model 5** | 0.78 | 0.68, 0.89 | <0.001 |  | 0.86 | 0.80, 0.93 | <0.001 |

**S5E Table. Hazard ratio for 5- and 10-year risk of death modelled against HDL cholesterol (multiple imputation).**

|  | **5 year risk** | | |  | **10 year risk** | | |
| --- | --- | --- | --- | --- | --- | --- | --- |
|  | **HR** | **95% CI** | **p-value** |  | **HR** | **95% CI** | **p-value** |
| **Model 1** | 0.76 | 0.50, 1.14 | 0.186 |  | 0.72 | 0.57, 0.92 | 0.008 |
| **Model 2** | 0.82 | 0.52, 1.29 | 0.384 |  | 0.80 | 0.62, 1.03 | 0.083 |
| **Model 3** | 0.64 | 0.43, 0.95 | 0.026 |  | 0.59 | 0.47, 0.74 | <0.001 |
| **Model 4** | 0.84 | 0.66, 1.27 | 0.408 |  | 0.80 | 0.63, 1.01 | 0.064 |
| **Model 5** | 0.73 | 0.48, 1.11 | 0.145 |  | 0.69 | 0.54, 0.87 | 0.002 |

**S5F Table. Hazard ratio for 5- and 10-year risk of death modelled against glycated haemoglobin (HbA1c) (multiple imputation).**

|  | **5 year risk** | | |  | **10 year risk** | | |
| --- | --- | --- | --- | --- | --- | --- | --- |
|  | **HR** | **95% CI** | **p-value** |  | **HR** | **95% CI** | **p-value** |
| **Model 1** | 1.48 | 1.34, 1.62 | <0.001 |  | 1.48 | 1.40, 1.57 | <0.001 |
| **Model 2** | 1.48 | 1.35, 1.63 | <0.001 |  | 1.49 | 1.41, 1.58 | <0.001 |
| **Model 3** | 1.19 | 1.04, 1.37 | 0.011 |  | 1.20 | 1.10, 1.30 | <0.001 |
| **Model 4** | 1.44 | 1.31, 1.58 | <0.001 |  | 1.45 | 1.36, 1.53 | <0.001 |
| **Model 5** | 1.17 | 1.02, 1.34 | 0.029 |  | 1.18 | 1.08, 1.28 | <0.001 |

**S5G Table. Hazard ratio for 5- and 10-year risk of death modelled against waist:hip ratio (WHR) (multiple imputation).**

|  | **5 year risk** | | |  | **10 year risk** | | |
| --- | --- | --- | --- | --- | --- | --- | --- |
|  | **HR** | **95% CI** | **p-value** |  | **HR** | **95% CI** | **p-value** |
| **Model 1** | 73.31 | 16.17, 332.43 | <0.001 |  | 118.56 | 45.08, 311.80 | <0.001 |
| **Model 2** | 158.23 | 25.71, 973.94 | <0.001 |  | 247.07 | 77.81, 784.50 | <0.001 |
| **Model 3** | 5.46 | 1.09, 27.39 | 0.039 |  | 10.01 | 3.61, 27.78 | <0.001 |
| **Model 4** | 58.83 | 12.81, 270.07 | <0.001 |  | 96.70 | 36.57, 255.65 | <0.001 |
| **Model 5** | 3.16 | 0.42, 23.90 | 0.266 |  | 5.44 | 1.54, 19.20 | 0.008 |

**S5H Table. Hazard ratio for 5- and 10-year risk of death modelled against C-Reactive Protein (CRP) (multiple imputation).**

|  | **5 year risk** | | |  | **10 year risk** | | |
| --- | --- | --- | --- | --- | --- | --- | --- |
|  | **HR** | **95% CI** | **p-value** |  | **HR** | **95% CI** | **p-value** |
| **Model 1** | 1.04 | 1.03, 1.04 | <0.001 |  | 1.03 | 1.03, 1.04 | <0.001 |
| **Model 2** | 1.04 | 1.03, 1.04 | <0.001 |  | 1.03 | 1.03, 1.04 | <0.001 |
| **Model 3** | 1.02 | 1.02, 1.03 | <0.001 |  | 1.02 | 1.01, 1.02 | <0.001 |
| **Model 4** | 1.04 | 1.03, 1.04 | <0.001 |  | 1.03 | 1.02, 1.04 | <0.001 |
| **Model 5** | 1.02 | 1.02, 1.03 | <0.001 |  | 1.02 | 1.01, 1.02 | <0.001 |
